# Temporal Integration of Auditory Information Is Invariant to Temporal Grouping Cues^1^,^2^,^3^

**DOI:** 10.1523/ENEURO.0077-14.2015

**Published:** 2015-04-30

**Authors:** Andrew S.K. Liu, Joji Tsunada, Joshua I. Gold, Yale E. Cohen

**Affiliations:** 1 Bioengineering Graduate Group; 2 Department of Otorhinolaryngology, Perelman School of Medicine; 3 Department of Neuroscience, Perelman School of Medicine, University of Pennsylvania, Philadelphia, Pennsylvania 19104

**Keywords:** auditory cortex, decision-making, scene analysis, speed-accuracy tradeoff, streaming

## Abstract

Auditory perception depends on the temporal structure of incoming acoustic stimuli. Here, we examined whether a temporal manipulation that affects the perceptual grouping also affects the time dependence of decisions regarding those stimuli. We designed a novel discrimination task that required human listeners to decide whether a sequence of tone bursts was increasing or decreasing in frequency. We manipulated temporal perceptual-grouping cues by changing the time interval between the tone bursts, which led to listeners hearing the sequences as a single sound for short intervals or discrete sounds for longer intervals. Despite these strong perceptual differences, this manipulation did not affect the efficiency of how auditory information was integrated over time to form a decision. Instead, the grouping manipulation affected subjects’ speed−accuracy trade-offs. These results indicate that the temporal dynamics of evidence accumulation for auditory perceptual decisions can be invariant to manipulations that affect the perceptual grouping of the evidence.

## Abstract (precis)

Auditory perception depends on perceptual grouping cues, which relate to how the brain parses the auditory scene into distinct perceptual units, and auditory decisions, which relate to how the brain identifies a sound. These two processes are not independent; both rely on the temporal structure of the acoustic stimulus.

## Significance Statement

Auditory perception depends on perceptual grouping cues, which relate to how the brain parses the auditory scene into distinct perceptual units, and auditory decisions, which relate to how the brain identifies a sound. These two processes are not independent; both rely on the temporal structure of the acoustic stimulus. However, the effects of this temporal structure on perceptual grouping and decision-making are not known. Here, we combined psychophysical testing with computational modeling to test the interaction of temporal perceptual grouping cues with the temporal processes that underlie perceptual decision-making. We found that temporal grouping cues do not affect the efficiency by which sensory evidence is accumulated to form a decision. Instead, the grouping cues modulate a subject’s speed−accuracy trade-off.

## Introduction

Auditory perception depends on both perceptual grouping and decision-making. Perceptual grouping is a form of feature-based stimulus segmentation that determines whether acoustic events are grouped into a single sound or segregated into distinct sounds (Bregman, 1990). Auditory decision-making involves the brain’s interpretation of information within and across discrete stimuli to detect, discriminate, or identify their source or content.

Auditory perceptual grouping and decision-making each depend critically on the temporal structure of incoming acoustic events. For instance, when a person is walking, each step is a unique acoustic event, but our auditory system groups these events together to form a stream of footsteps. However, if the time between events is long, the auditory system segregates these events into unique, discrete sounds. Decision-making can also depend on the temporal structure of a sound because decision-making is a deliberative process in which listeners often accumulate and interpret auditory information over time to form categorical judgments (Green et al., 2010; Brunton et al., 2013; Mulder et al., 2013).

Although we know that perceptual grouping can affect some forms of decision-making (Bey and McAdams, 2002; Roberts et al., 2002; Micheyl and Oxenham, 2010; Borchert et al., 2011; Thompson et al., 2011), the interplay between the temporal properties of an auditory stimulus, perceptual grouping, and decision-making is not known. We cannot infer this interplay from visual studies because analogous manipulations in the visual domain (Kiani et al., 2013) do not relate directly to auditory perceptual grouping. Further, because temporal processing is fundamentally different for audition than for vision (Bregman, 1990; Griffiths and Warren, 2004; Shinn-Cunningham, 2008; Shamma et al., 2011; Bizley and Cohen, 2013), it is reasonable to hypothesize that this interplay may be different in these two sensory systems. Thus, it remains an open and fundamental question whether and how grouping temporal information interacts with auditory grouping and decision-making.

To examine this question, we measured the performance of human subjects who participated in a series of auditory tasks in which they reported whether a sequence of tone bursts was increasing or decreasing in frequency. Temporal information was manipulated by changing the interval between the onsets of consecutive tone bursts. This manipulation affected the subjects’ perceptual grouping of the tone-burst sequence: they heard one sound when the interval was short but a series of discrete sounds when it was long. The quality of the sensory evidence was manipulated by changing the proportion of tone bursts that linearly increased or decreased in frequency. We found that subjects accumulated sensory evidence over time to form their decisions. However, the time interval between consecutive tone bursts did not affect how this incoming stimulus was accumulated to form the decision about the change in frequency. Instead, the time between the tone bursts affected how the subjects balanced the speed and accuracy of their decisions, which fundamentally trade-off for certain decisions like this one that require incoming, noisy information to be accumulated over time (Gold and Shadlen, 2007; Bogacz et al., 2010). Specifically, for our task, longer time intervals between tone bursts (i.e., slower rates of incoming sensory information) led to a higher premium on speed at the expense of accuracy. Overall, these findings indicate that temporal manipulations that affect the perceptual grouping of sounds do not necessarily affect how information from those sounds are accumulated over time to form a decision, even when the temporal manipulations have clear effects on the trade-off between the speed and accuracy of the decision.

## Materials and Methods

Prior to their participation, subjects provided informed consent. Human subjects were recruited at the University of Pennsylvania. All subjects (age range: 25-48 years) reported normal hearing; three of the subjects were authors on the study.

### Experimental setup

All experimental sessions took place in a single-walled acoustic chamber (Industrial Acoustics) that was lined with echo-absorbing foam. Each subject was seated with his or her chin in a chin rest that was approximately 2 ft from a calibrated Yamaha (model MSP7) speaker. Auditory stimuli were generated using the RX6 digital-signal-processing platform (TDT Inc.). The task structure was controlled through the Snow Dots toolbox (http://code.google.com/p/snow-dots) that ran in the Matlab (Mathworks) programming environment. Subjects indicated their responses by pressing a button on a gamepad (Microsoft Sidewinder). Tasks instructions and feedback were presented on a LCD flat-panel monitor (Dell E171FP) that was placed above the speaker.

### Auditory stimuli

The auditory stimulus was a sequence of tone bursts (duration: 30 ms with a 5 ms cos^2^ gate; level: 65 dB SPL). The interburst interval (IBI) was the time between the offset of one tone burst and the onset of the next tone burst (range: 10-150 ms).

At the beginning of each trial, the frequency of a sequence’s first tone burst was randomly sampled from a uniform distribution (500-3500 Hz). Next, we generated a monotonically increasing or decreasing sequence by adding or subtracting a 7.5 Hz to the previous tone-burst frequency. Finally, on a trial-by-trial basis, we perturbed the temporal order of the tone bursts. This perturbation changed the quality of the sensory evidence. In particular, for each trial, we defined a sequence’s coherence, which was the proportion of tone bursts in a sequence whose frequency value changed by a fixed increment relative to the previous tone burst. If the coherence was 100%, all of the tone bursts monotonically increased or decreased. If the coherence was <100%, the temporal order of a subset of tone bursts was randomly shuffled. For example, if the coherence was 50%, half of the tone bursts were shuffled. If the coherence was 0%, all of the tone bursts in the sequence were shuffled.

This stimulus design ensured that the frequency range of the sequences overlapped and that, on average, each sequence contained the same frequency values (but in a different temporal order). This procedure also minimized the possibility that subjects based their decisions on the specific frequency content of a sequence rather than its sequence direction.

**Figure 1:**
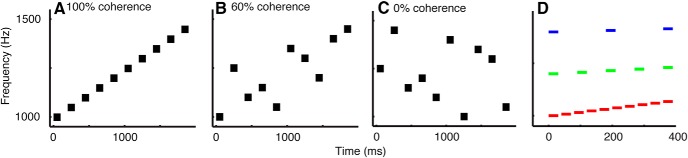
Example auditory sequences characterized in terms of coherence and different interburst intervals. ***A***, Frequency direction (i.e., increasing) is easiest to discriminate when the stimulus is 100% coherent. As coherence gets smaller (60% in ***B*** and 0% in ***C***), frequency direction becomes more ambiguous and, thus, can lead to more errors. In ***A***−***C***, we show increasing auditory sequences. Decreasing sequences are analogous but with negative coherence values. ***D*** shows a 100% coherence auditory sequence at three different IBIs: 10 ms (red), 60 ms (green), and 150 ms (blue).

Overall, each tone-burst sequence could be characterized by three parameters (Fig. 1): (1) direction, which indicated whether a sequence of tone bursts increased or decreased in frequency; (2) IBI; and (3) coherence. The values of each of these three parameters were determined on a trial-by-trial basis depending on the constraints of each auditory task; see Behavioral tasks, below.

### Behavioral tasks

Subjects participated in three versions of the discrimination task. For all three versions, subjects reported the direction (increasing or decreasing) of the tone-burst sequence and received visual feedback, via the LCD monitor, regarding their report on every trial. The time between trials was ∼2 s and was independent of the subjects’ report.

### Response-time task

**Figure 2:**
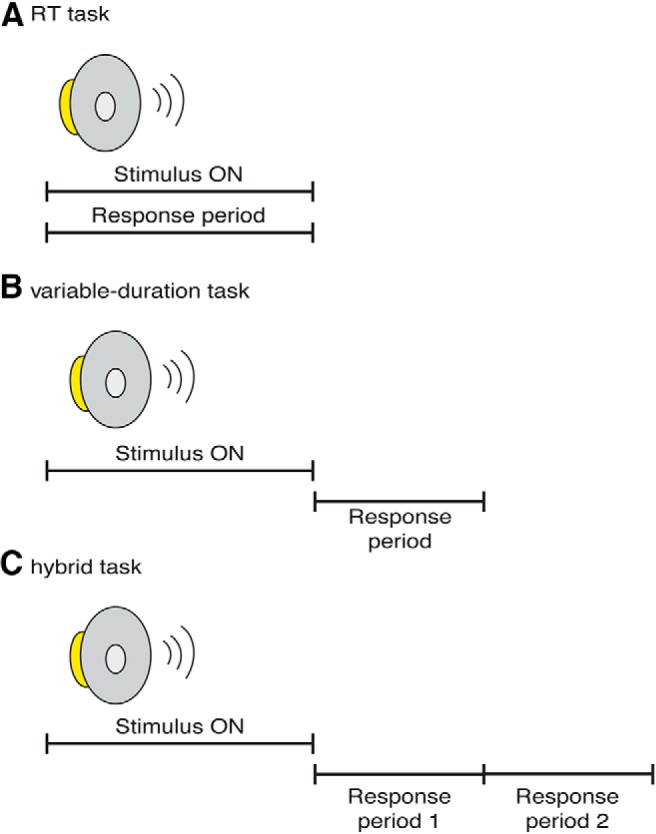
Task design. ***A***, For the RT task, subjects indicated their choice (i.e., sequence direction) any time after onset of the sequence. ***B***, For the variable-duration task, subjects indicated their choice after sequence offset. ***C***, For the hybrid task, subjects indicated sequence direction and whether they heard the sequence as one sound or discrete sounds after sequence offset in separate response periods. For all three tasks, subjects were provided feedback regarding frequency direction at the end of each trial (not shown).

For this task, subjects reported their perceptual decision at any time following sequence onset (Fig. 2*A*). They were instructed to respond as quickly as possible but not to sacrifice accuracy. We tested six subjects (5 male and 1 female) in four weekly 1.5 h sessions. Each session contained four blocks of trials; a short break was provided between blocks. In each block, we varied sequence direction (increasing or decreasing), IBI (10, 60, or 150 ms), and coherence (0, 10, 25, 50, or 100%) on a trial-by-trial basis. Each combination of these sequence parameters was presented five times within a block for a total of 150 trials/block. The maximum response-time (RT) (and, hence, maximum sequence duration) was 5000 ms; a trial was aborted if a subject did not respond by the end of the sequence. The stimulus was terminated as soon as the subject reported the decision.

Four subjects participated in additional sessions (four blocks of trials in each of four sessions per subject) of the RT task to test for effects of changes in the speed−accuracy trade-off. For these sessions, the IBI was held constant at 60 ms and subjects were instructed to “emphasize speed” or “emphasize accuracy” in alternating blocks. Stimulus tone-sequence direction (increasing or decreasing) and coherence (0, 10, 25, 50, or 100%) were varied on a trial-by-trial basis.

### Variable-duration task

For this task, the experimenter controlled listening duration: subjects reported their direction decision following offset of the auditory sequence (Fig. 2*B*). Five of the six subjects tested in the RT task (4 male and 1 female) also participated in six weekly 1.5 h sessions of the variable-duration task. On each trial, we chose the duration of the sequence by sampling from a truncated exponential distribution (rate parameter = 2000 ms for all IBIs); choosing the sequence duration from this distribution minimized the possibility that subjects could anticipate the end of the sequence (Gold and Shadlen, 2003). The upper- and lower-stimulus durations were a function of IBI: for IBI = 10 ms, min = 160, max = 1400 ms; for IBI = 60 ms, min = 360, max = 3150 ms; and for IBI = 150 ms, min = 720, max = 6300 ms. These limits were chosen so that each sequence, independent of IBI, contained 4-35 tone bursts. At the end of each sequence, a response cue was flashed on the LCD monitor and subjects had 800 ms to respond. Each session contained four blocks of trials; a short break was provided between blocks. In each block, we varied sequence direction (increasing or decreasing), IBI (10, 60, or 150 ms), and coherence (0, 10, 25, 50, or 100%) on a trial-by-trial basis. Each combination of these sequence parameters was presented five times within a block, for a total of 150 trials per block.

### Hybrid task

For this task, subjects participated in a version of the variable-duration task that also required them to report, on each trial, whether they heard the sequence as one sound or as a series of discrete sounds (Fig. 2*C*). Four of the six subjects tested in the RT task plus one new subject (3 male and 2 female) participated in four weekly 1.25 h sessions of the hybrid task. Sequence duration was sampled from a truncated exponential distribution and the limits of the stimulus durations were set to ensure that each sequence had 4-35 tone bursts. Subjects reported their two decisions during two separate 800 ms response periods. Prior to each sequence’s onset, a colored cue, which was presented on the LCD monitor, indicated the temporal order in which subjects were to report their decisions; this order alternated on a block-by-block basis.

For this task, we set both IBI and coherence to values that were centered on each subject’s psychophysical threshold. IBI threshold was the IBI value rated as one sound 50% of the time. Because the subjects’ 50%-IBI threshold varied on a day-by-day basis, we measured this threshold daily, prior to their participation in the hybrid task. IBI threshold was measured using a one-up/one-down adaptive procedure (Treutwein, 1995; García-Pérez, 1998). The sequence always had 16 tone bursts and used a 50% coherence stimulus.

Coherence threshold was defined to be 70.7% correct performance, which corresponds to a d′ of 0.77. Because preliminary experiments indicated that coherence threshold was constant across experimental sessions, it was measured once for each subject. Coherence threshold was calculated using a two-up/one-down adaptive procedure (Treutwein, 1995; García-Pérez, 1998). During this procedure, the sequence’s IBI was set to the IBI threshold.

For the hybrid task, each session contained four blocks of trials; a short break was provided between blocks. We varied IBI, on a trial-by-trial basis, between three different values: (1) IBI threshold minus 15 ms (20 trials per block), (2) IBI threshold (80 trials per block), and (3) IBI threshold plus 15 ms (20 trials per block). Coherence was set to each subject’s coherence threshold.

### Fitting of behavioral data to sequential-sampling models

Behavioral data were fit to variants of sequential-sampling models related to the drift diffusion model (DDM) (Ratcliff et al., 2004; Smith and Ratcliff, 2004; Gold and Shadlen, 2007; Green et al., 2010; Brunton et al., 2013; Mulder et al., 2013) to quantify the effects of sequence coherence and IBI on the decision-making process. These models describe the process of converting incoming sensory evidence, which is represented in the brain as the noisy spiking activity of populations of relevant sensory neurons, into a decision variable that can guide behavior.

### RT task

For the RT task, a key benefit of these sequential-sampling models is that they make quantitative predictions about both choice and RT as a function of the coherence of the auditory sequence. In other words, these models simultaneously fit: (1) the psychometric function, which describes accuracy versus sequence coherence; and (2) the chronometric function, which describes RT versus sequence coherence. We used several model variants.

#### Model variant #1

The first variant was a standard, symmetric DDM in which a perceptual decision is based on an accumulation over time of noisy evidence to a fixed bound, a process that is mathematically equivalent to the one-dimensional movement of a particle undergoing Brownian motion to a boundary (Ratcliff et al., 1999; Gold and Shadlen, 2002; Shadlen et al., 2006; Eckhoff et al., 2008; Green et al., 2010; Ding and Gold, 2012; Brunton et al., 2013; Mulder et al., 2013). In brief, this version had seven free parameters: one drift rate (*k*) per IBI; one symmetric bound for either increasing or decreasing (+*A* or −*A*; i.e., the height of the bound was the same for both choices but with an opposite sign) choices per IBI; and a single non-decision time (*T*_ND_) that accounts for sensory-processing and motor-preparation time. Drift rate governs sensitivity and is implemented in terms of the moment-by-moment sensory evidence, which has a Gaussian distribution *N*(µ,1) with a mean µ that scales with sequence coherence (*C*): µ = *kC*. The decision variable is the temporal accumulation of this momentary sensory evidence. A decision (i.e., the subject reports that the sequence is increasing or decreasing) occurs when this decision variable reaches a decision bound (+*A* or −*A*, respectively). The decision time is operationally defined as the time between auditory-sequence onset and the cross of either bound. RT is the sum of the decision time and the associated non-decision time. The probability that the decision variable first crosses the +*A* bound is $\frac{{\text{e}}^{2\text{μA}}\,\;-\;1}{{\text{e}}^{2\text{μA}}\,\;-\;{\text{e}}^{-2\text{μA}}}$. The mean decision time is $\frac{2\text{A}}{\text{μ}}\coth\left(2\text{μA}\right)-\frac{\text{A}}{\text{μ}}\coth\left(\text{μA}\right)$ for increasing choices and $\frac{2\text{A}}{\text{μ}}\coth\left(2\text{μA}\right)-\frac{\text{A}}{\text{μ}}\coth\left(\text{μA}\right)$ for decreasing choices.

#### Model variant #2

The second variant was a leaky accumulator, in the form of a stable Ornstein−Uhlenbeck process (Busemeyer and Townsend, 1993; Bogacz et al., 2006), in which positive values of the leak term imply that a given decision is influenced most strongly by the most recent samples of sensory evidence. Because these processes do not have simple, exact analytic solutions for both the psychometric and chronometric functions, we conducted simulations to fit the data. For each fit, we simulated 40,000 trials per iteration, with the decision variable computed in 1 ms steps.

#### Model variant #3

The final variant was an accumulate-to-bound model with non-leaky drift and bounds that can collapse (decrease towards zero) as a function of time within a trial (Ditterich, 2006b; Drugowitsch et al., 2012; Thura et al., 2012; Hawkins et al., 2015). The dynamics of the collapsing process were governed by a two-parameter Weibull function (Hawkins et al., 2015). These fits were also obtained using simulations like the leaky-accumulator model, above.

All model fits were computed by first using a pattern-search algorithm to find suitable initial conditions (“patternsearch” in the Matlab programming environment) and then a gradient descent to find the best-fitting parameters (“fmincon” in Matlab) that minimized the negative log-likelihood of the data, given the model fits (Palmer et al., 2005). The likelihood function for subjects’ choices was modeled as binomial errors and the mean response times were modeled as Gaussian errors.

### Variable-duration and hybrid tasks

For these tasks, the experimenter, not the subject, controlled listening duration. Therefore, we used models that had a different stopping rule than those used for the RT tasks. Specifically, these models assumed that the decision was made based on the sign of the accumulated evidence at the end of the stimulus presentation. These models included two basic parameters: (1) a coherence-scaling term (*k*), which governed the relationship between sequence coherence and the strength of the evidence to accumulate; and (2) accumulation leak (λ), which governed the efficiency of the accumulation process. We computed the probability (*p*) of a correct response as a function of sequence coherence (*C*) and listening duration (*D*) as: $\text{p}\left(\text{C},\text{D}\right)=0.5\,\;*\;\left(1-(1-L)\;*\;\varphi \left(-\text{kC}\sqrt{\frac{2\left({\text{e}}^{\text{λD}}\,\;-\;1\right)}{\text{λ}\left({\text{e}}^{\text{λD}}\,\;+\;1\right)}}\right)\right)$, where φ is the normal standard cumulative distribution function. The lapse rate (*L*) was set to a small value (0.01) to provide better fits (Klein, 2001; Wichmann and Hill, 2001). The models were fit using Matlab’s “fmincon” function to minimize the log-likelihood of the data, given the parameters, and assuming binomial errors.

### Fitting of RT task data to the LATER model

RT distributions from the RT task were also analyzed using the LATER (Linear Approach to Threshold in Ergodic Rate) model. This model assumes that RT distributions are distributed as an inverse Gaussian because they result from a process with a linear rate of rise, which are distributed across trials as a Gaussian, that trigger a movement when reaching a fixed threshold (Carpenter and Williams, 1995; Reddi et al., 2003). We used maximum-likelihood methods to fit RT distributions to a model with two free parameters that represented the mean rate-of-rise and the threshold. We tested if and how each parameter varied with IBI for each subject, direction, and stimulus coherence.

### Calculating psychophysical kernels from the RT data

Finally, we calculated the subjects’ psychophysical kernels from the RT task to further support the idea that the subjects were using a process akin to bounded accumulation. The kernels were computed directly from the data by taking, from each 0% coherence trial, the mean-subtracted stimulus sequence and then computing the mean (and standard error) value of these time-dependent sequences separately for trials leading to increasing or decreasing choices. We fit these kernels to a model that sorted the 0%-coherence trials into two categories based on the sign of the slope of a linear regression of frequency versus burst number. We used a grid search to find the values of two parameters—one governing the number of tone bursts from stimulus onset that is used to compute the linear regression and a second that scales the stimulus-frequency values used to compute the kernel—that maximized the likelihood of obtaining the IBI-specific kernels measured from the data given the model.

## Results

We used human psychophysics to test if and how the time course of evidence accumulation for an auditory-discrimination task was affected by a temporal manipulation that modified the perceptual grouping of the sensory evidence. The task required subjects to report whether the frequency direction of a tone-burst sequence was increasing or decreasing (Fig. 1*A*). Task difficulty was manipulated by controlling the coherence of the sequence, which corresponded to the fraction of tone bursts whose frequencies increased or decreased systematically (Fig. 1*A−C*).

**Figure 3:**
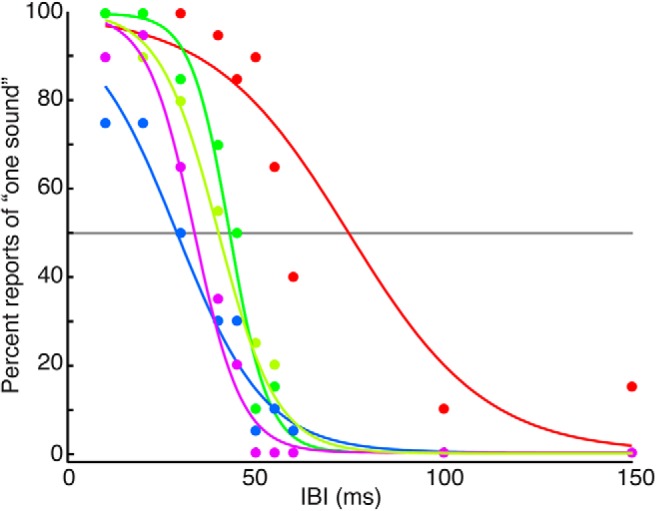
Influence of IBI on reports of perceived grouping. Subjects reported whether they perceived the stimulus as one sound or discrete sounds. The graph shows proportion of trials in which each subject chose one sound as a function of IBI. The points indicate each subject’s performance. Each curve represents a logistic function that was fit to each subject’s reports across four sessions. The gray line indicates 50% of trials reported as “one sound”, which was IBI threshold. Colors represent different subjects.

The temporal manipulation was a change in the IBI of the stimulus sequence, which affected perceptual grouping. For short IBIs (<∼30 ms), subjects tended to report that the sequence was one sound. For medium IBIs (∼30-100 ms), subjects alternated trial-by-trial between reports that the sequence was one sound or a series of discrete sounds. For long IBIs (>∼100 ms), subjects reliably reported that the sequence was a series of discrete sounds (Fig. 3). To test how this grouping manipulation affected the temporal dynamics of the perceptual decision (i.e., the frequency direction of the tone-burst sequence), we used three versions of a discrimination task: an RT task in which subjects controlled listening duration; a variable-duration task in which the experimenter controlled listening duration; and a hybrid task that combined the variable-duration task with an explicit grouping judgment about whether the sound was one sound or a series of discrete sounds. Results from each task are presented below.

### Response time task

**Figure 4:**
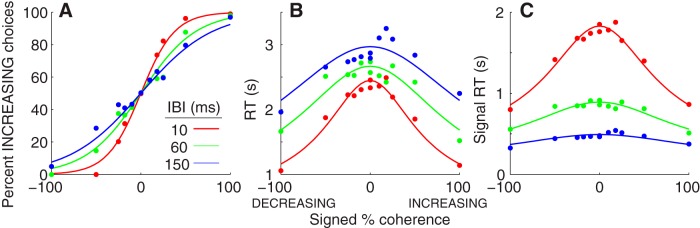
Performance on the RT task. ***A*,** The fraction of trials in which subjects reported a sequence of tone bursts was increasing in frequency as a function of signed coherence and IBI. Positive coherence values indicate that the sequence was increasing in frequency; negative indicates decreasing. ***B***, The mean RT (i.e., the time between sequence onset and button press) as a function of signed coherence and IBI on correct trials only (and all 0% coherence trials). ***C***, As in ***B***, but using signal RT (i.e., RT not including cumulative IBI). The solid curves are simultaneous fits of psychometric and chronometric data to a DDM (see Results). The psychometric data (***A***) only show the fit to the signal-RT data (***C***). In each panel, the points indicate performance data that was pooled across subjects. Colors represent different IBIs, as indicated in ***A***.

Both accuracy and RT depended systematically on coherence and IBI. Figure 4 summarizes the behavioral performance of all six subjects. Choices tended to be more accurate and faster when stimulus coherence was high than when it was low.

Subjects’ choices were clearly based on the direction of frequency change and not simply the absolute frequency value of the sequence. Specifically, when we analyzed behavior relative to the beginning, mean, and ending frequency value of the sequence, performance was near chance: the median (full range) percent correct of all six subjects’ choices, when collapsed across coherences and IBIs, was 56.9% (55.3-58.0), 50.6% (50.2-51.9), and 55.4% (54.6-56.6), respectively.

We also found that the relationships between choice, RT, and coherence were modulated by IBI. Longer IBIs led to shallower psychometric functions (i.e., lower sensitivity; Fig. 4*A*) and longer RTs (Fig. 4*B*). When we considered the portion of the RT that only included presentation of the tone bursts and not the silent periods by subtracting out the cumulative IBIs (which we refer to as “signal RT” and which treats the cumulative IBIs, like sensory and motor processing, as part of the non-decision time on a given trial), the effects of IBI on RT were reversed: the longest signal RTs corresponded to the shortest IBIs (Fig. 4*C*).

We quantified the effects of coherence and IBI on the time-dependent decision process by fitting the choice and RT data for each subject and from pooled data across subjects to several variants of models that are related to the DDM. All of the models had the same basic form. They assumed that the decision was based on the temporal accumulation of noisy evidence until reaching one of two prespecified boundaries. The signal-to-noise ratio of this decision variable was governed by a drift rate, which was proportional to stimulus coherence and, in some cases, was also subject to leaky accumulation (Busemeyer and Townsend, 1993; Usher and McClelland, 2001; Tsetsos et al., 2012). Choice was governed by the identity of the reached boundary, which, in some cases, could change as a function of time within a trial to reflect an increasing urgency to respond (Ditterich, 2006b; Drugowitsch et al., 2012; Thura et al., 2012; Hawkins et al., 2015). RT was governed by the time to reach the boundary plus extra non-decision time. The height of the boundary governed the trade-off between speed and accuracy: higher boundaries provided longer decision times and higher accuracy, whereas lower boundaries increased speed at the expense of accuracy (Gold and Shadlen, 2007). Because performance tended to be symmetric for the two choices [across all subjects and IBIs and using balanced stimulus presentations, the median (interquartile range) absolute value of the difference in the fraction of increasing vs decreasing choices was only 0.001 (0.000–0.004)], we used an unbiased model with seven parameters: one non-decision time, three parameters representing drift rate per each of the three IBI conditions, and three parameters representing the bound height per IBI.

Fits of this model to choice data and either raw or signal RTs were consistent with a decision variable that was based on the signal portion of the stimulus sequence but not the time between bursts (the IBI). Drift rate has units of change of standardized evidence per unit time. Therefore, converting from raw to signal RT affects the (linear) scaling of this value, which is governed by the duty cycle associated with the given IBI: *b*/(IBI + *b*), where *b* is burst duration of 30 ms. Accordingly, best-fitting values of the DDM parameters that were fit to choices and raw RT were scaled versions of those fit to choices and signal RTs, with the scale factors approximately equal to the IBI-specific duty cycles [slopes of linear regressions of subject-specific signal vs raw drift rates = 0.80 (duty cycle = 0.75), 0.43 (0.33), and 0.23 (0.17) for IBI = 10, 60, and 150 ms, respectively]. Furthermore, best-fitting signal drift rates that were rescaled and expressed in units of the change in evidence per unit of raw time (i.e., multiplied by the duty cycle) were strongly correlated with the associated, best-fitting raw drift rates across IBIs and subjects (*r* = 0.98, *p* < 0.01^a^; Table 1). These results imply that the IBI manipulation affected only the duty-cycle-dependent scaling of drift rates. Therefore, we used signal RTs for the model fits, which corresponded to drift rates that had the same temporal scaling and thus could be compared directly across IBI conditions.

**Table 1:** Statistical table

	Data structure	Statistical test	Power
a	Normal distribution	Pearson Correlation	*p* < 0.01
b	Normal distribution	Likelihood-ratio test; Bonferroni-corrected for three parameters	*p* < 0.001
c	Normal distribution	Likelihood-ratio test; Bonferroni-corrected for three parameters	*p* < 0.01
d	Normal distribution	Likelihood-ratio test	*p* > 0.24
e	Normal distribution	Likelihood-ratio test	*p* > 0.1
f	Normality not assumed	Mann−Whitney test	*p* < 0.01
g	Normality not assumed	Kruskal−Wallis test	*p* < 0.001
h	Normal distribution	Likelihood-ratio test, Bonferroni-corrected for two parameters	*p* < 0.01
i	Normality not assumed	Kruskal−Wallis test	*p* > 0.05
j	Normal distribution	Likelihood-ratio test, Bonferroni-corrected for two parameters	*p* < 0.01
k	Normal distribution	Likelihood-ratio test	*p* = 0.2838
l	Normality not assumed	Kruskal−Wallis test	*p* = 0.011
m	Normality not assumed	Kruskal−Wallis test	*p* = 0.125
n	Normal distribution	Likelihood-ratio test	*p* > 0.05
o	Normality not assumed	Kruskal−Wallis test	*p* > 0.05
p	Normality not assumed	Mann−Whitney test	*p* < 0.05
q	Normal distribution	Likelihood-ratio test, Bonferroni-corrected for two parameters	*p* < 0.01
r	Normal distribution	Likelihood-ratio test, Bonferroni-corrected for two parameters	*p* < 0.01

**Figure 5:**
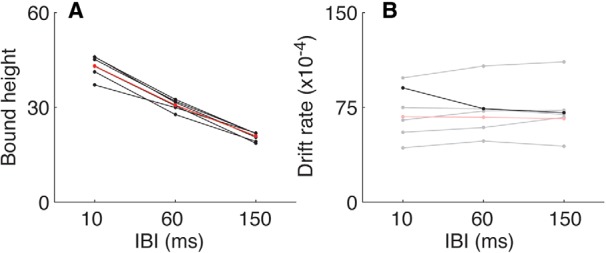
Parameter values from fits of the basic DDM to the RT-task data. Each panel shows best-fitting values of bound height (***A***) or drift rate (***B***) plotted as a function of IBI for fits to data from individual subjects (black) or combined across all subjects (red). Dark lines/symbols indicate that the model fits were improved significantly by fitting the given parameter separately for each IBI condition (likelihood-ratio test, *p* < 0.01, Bonferroni-corrected for three parameters).

We found that the effects of IBI on choice and signal RT primarily reflected changes in the decision boundary but not the drift rate (Fig. 5). The height of a symmetric, fixed decision boundary (i.e., the same height for both increasing and decreasing choices) declined systematically with increasing IBI for all six subjects and for data combined across subjects (likelihood-ratio test comparing a seven-parameter model with separate values of drift rate and bound height per IBI plus a non-decision time to a five-parameter model with a single value of drift rate shared across IBIs, *p* < 0.001^b^ in all cases; Bonferroni-corrected for three parameters; Fig. 5*A*). In contrast, drift rate depended on IBI for only one of the six subjects (*p* < 0.01^c^; Bonferroni-corrected for three parameters) and not for the other subjects or combined data (Fig. 5*B*). These model fits were not improved by adding to the model either leaky accumulation (likelihood-ratio test, *p* > 0.24^d^ across subjects and for all data combined) or collapsing bounds (likelihood-ratio test, *p* > 0.1^e^ for five of the six subjects and for all data combined). There also was little evidence for slow errors that can be expected in models with collapsing bounds, with only eight of 216 conditions separated by subject/coherence/IBI showing such an effect (Mann−Whitney test comparing median correct vs error RTs, *p* < 0.01; Ditterich, 2006a)^f^. Likewise, there was little evidence for fast errors that can be expected in models with variable bounds, with only three conditions showing such an effect (Ratcliff and Rouder, 1998). Thus, changes in IBI, which affected perceptual grouping and the rate of arrival of decision-relevant signals, caused systematic, robust changes in the speed−accuracy trade-off governed by a fixed, time-independent bound. In contrast, the changes in IBI did not cause systematic changes in the efficiency with which sensory evidence was accumulated over time to form the decision.

**Figure 6:**
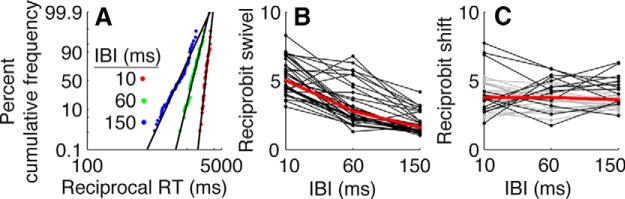
LATER model fits to signal RT data. ***A***, Distributions of signal RT from 0%-coherence trials for one subject are plotted on a reciprobit plot: reciprocal RT versus percentage of cumulative frequency on a probit scale (Reddi et al., 2003), separately per IBI. Best-fitting values of the bound height (***B***) and mean rate-of-rise (***C***) of the LATER model (see Results and Materials and Methods for details) are plotted as a function of IBI for each subject and coherence (black/gray lines and data points). The data in black indicate that the model fits were improved significantly by fitting the given parameter separately for each IBI condition (likelihood-ratio test, *p* < 0.01, Bonferroni-corrected for two parameters). Shaded lines/symbols indicate that the model fits were not improved significantly. Red data points/lines represent the median values across all conditions.

These results were supported by independent analyses of the signal-RT distributions. A useful way to assess possible changes in drift rate and/or bound height in a simple accumulate-to-bound framework is to use the LATER model (Carpenter and Williams, 1995; Reddi et al., 2003). According to this model, a decision variable rises linearly to a threshold (bound) in order to trigger a motor response. Assuming a fixed bound, but a noisy decision variable with a rate of rise that is normally distributed across trials, RT is distributed as an inverse Gaussian. This distribution can be plotted as a straight line on “reciprobit” axes (i.e., percent cumulative frequency on a probit scale vs the reciprocal of RT from 0%-coherence trials; Fig. 6*A*). Horizontal shifts of these lines imply changes in the mean rate-of-rise of the decision variable, whereas swivels about a fixed point at infinite RT imply changes in the bound height (Reddi et al., 2003). When we fit the LATER model to signal-RT data separately for each subject, coherence, and IBI (correct trials only), we found that increasing IBI caused systematic decreases in the bound (Kruskal−Wallis test for *H*_0_: equal median values per IBI, across subjects and coherences, *p* < 0.001^g^; 34 of 36 individual subject−coherence pairs had a significant dependence of bound height on IBI, all of which had a lower bound for the longest vs the shortest IBI, *p* < 0.01^h^, likelihood-ratio test, Bonferroni-corrected for two parameters; Fig. 6*B*). In contrast, increasing IBI did not cause a systematic change in the best-fitting mean rate-of-rise (Kruskal−Wallis test, *p* > 0.05^i^; 13 of 36 individual subject−coherence pairs had a significant dependence of rate-of-rise on IBI, of which seven showed an increasing rate-of-rise and six showed a decreasing rate-of-rise; *p* < 0.01^j^, likelihood-ratio test, Bonferroni-corrected for two parameters; Fig. 6*C*).

**Figure 7:**
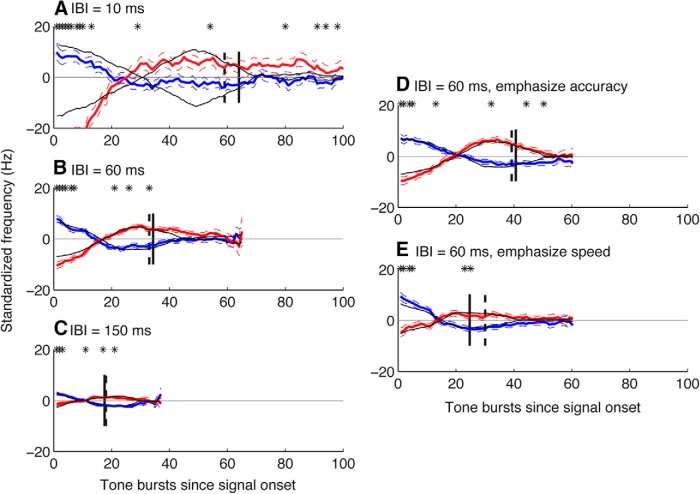
Psychophysical kernels. Kernels represent the average of the stimulus sequence (mean subtracted on each trial) presented on all 0%-coherence trials across subjects for increasing (red) and decreasing (blue) choices. ***A−C***, Kernels computed per IBI condition, as indicated, and smoothed using a 21-sample moving mean. ***D***, ***E***, Kernels for the 60 ms IBI condition when subjects were told to emphasize accuracy or speed, respectively. Thick and broken lines are mean and standard error, respectively. Data are aligned relative to onset of the sequence. Asterisks indicate that the increasing and decreasing kernels were significantly different from one another for a given time bin (Mann−Whitney test, *p* < 0.05^p^, using the raw, unsmoothed kernels). Black lines indicate best-fitting simulated kernels (see text for details). Solid vertical lines indicate median RT. Dashed vertical lines indicate the end of the integration time from the best-fitting simulated kernels.

These results were also supported by correlation analyses that related choices to the noisy auditory stimulus (Knoblauch and Maloney, 2008; Murray, 2012). We computed two kernels per IBI condition, one for increasing choices and the other for decreasing choices, from the 0%-coherence trials from all six subjects. Each kernel represented the mean, within-trial time course of the mean-subtracted, stochastic auditory sequence that led to the given choice (Fig. 7). Subjects made increasing choices when the frequency tended to increase throughout most of the trial, with average kernels that started below the within-trial mean, then increased steadily to a peak value above the within-trial mean around the time of the median RT, then reverted back towards the mean. Likewise, subjects made decreasing choices on trials in which the frequency progression of the stochastic stimulus moved in the opposite direction, starting relatively high and then decreasing for much of the trial.

These kernels were consistent with a DDM-like decision process that had lower bounds for longer IBIs, corresponding to an increasing emphasis on speed at the expense of accuracy. Specifically, the choice selectivity of these kernels (i.e., the time bins in which increasing and decreasing kernels differed from each other), measured in terms of tone bursts within a stimulus sequence and thus consistent with the signal RT analyses described above, was longest for the shortest IBI and shortest for the longest IBI (compare asterisks in Fig. 7*A−C*). We found a similar effect when using just the 60 ms IBI but providing explicit instructions to the subjects about the speed−accuracy trade-off, with relatively longer choice selectivity under an “emphasize accuracy” condition (Fig. 7*D*) and relatively shorter choice selectivity under an “emphasize speed” condition (Fig. 7*E*). These kernels were also qualitatively consistent with an evidence-accumulation process with little or no leak because choice selectivity was strongest at the beginning of a trial; if leak was present, it would tend to show up as less choice selectivity at the beginning of a trial.

To more quantitatively relate these kernels to the underlying decision process, we fit them to a simple, two-parameter model that assumed that choices were based on particular frequency progressions within the given stimulus. One parameter governed the time course of the relevant progression, which could range from just the first two bursts to the full sequence; i.e., simulated increasing or decreasing choices occurred when the slope of a linear regression of frequency versus burst number for the first *n* bursts in a sequence was >0 or <0, respectively. The second parameter scaled the contribution of each stimulus sequence to the final kernel, akin to the signal-to-noise ratio (SNR) of the internal stimulus representation. We found that the best-fitting value of the integration time decreased systematically with increasing IBI (and for the “emphasize speed” relative to the “emphasize accuracy” instruction), in each case closely matching the IBI-specific median RTs (compare solid and dashed vertical lines in Fig. 7). In contrast, the best-fitting scale factor did not differ as a function of IBI (likelihood-ratio test, *p* = 0.28^k^), implying a consistent SNR across conditions (like the IBI-independent drift rate in Fig. 5). Thus, like the DDM- and LATER-based analyses described above, these kernel analyses implied that the IBI manipulation affected the speed−accuracy trade-off on the RT task but not how information was accumulated over time to form the decision.

### Variable-duration task

Because the psychometric and chronometric data from the RT task depended on each subject’s individual speed−accuracy tradeoff, it is possible that the DDM-model fits reflected complex interactions between the rate of sensory-evidence accumulation and bound heights across IBI conditions (Ratcliff and Tuerlinckx, 2002). To directly test the relationship between IBI and the rate of evidence accumulation, subjects participated in the variable-duration task. During this task, we experimentally controlled the duration of the auditory sequence and, hence, the amount of sensory evidence (Fig. 2*B*). Analogous to the RT task, we analyzed performance as a function of signal time to standardize the sequence duration with respect to the rate of tone-burst presentation.

**Figure 8:**
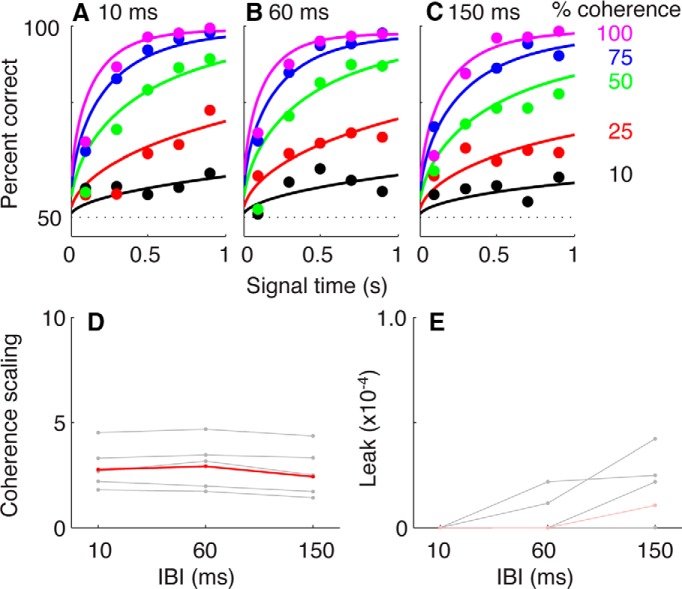
Performance on the variable-duration task. ***A−C***, Psychometric data are plotted as a function of listening duration for different coherences and IBIs, as indicated. Each data point reflects mean performance for all five subjects as a function of coherence and signal time (plotted in 0.2 s bins, up to 1.0 s, but fit using unbinned data). The solid curves are fits from the best-fitting model with two parameters: drift rate and accumulation leak. ***D***, ***E***, Best-fitting values of drift rate (***D***) and accumulation leak (***E***) plotted as a function of IBI for fits to data from individual subjects (black) or combined across all subjects (red). Dark lines and symbols indicate that the model fits were improved significantly by fitting the given parameter separately for each IBI condition (likelihood-ratio test, *p* < 0.01^q^, Bonferroni-corrected for two parameters). Shaded lines and symbols indicate that the model fits were not improved significantly.

Mean performance accuracy for all five subjects improved systematically as a function of both coherence and signal time, in a manner that was qualitatively similar for all three IBI conditions (Fig. 8*A−C*). For each condition, accuracy tended to reach an upper asymptote of >99% correct in <1000 ms of signal time for the highest coherences; accuracy rose steadily at longer listening times for lower coherences.

We again quantified these effects by fitting the choice data to DDM-like models (Fig. 8*D*,*E*). Like the DDM described in the Response time task section (above), all of these models assumed that the decision was based on the value of a decision variable, which represented the accumulation of noisy sensory evidence over time. Like the RT fits, we assumed a drift rate that scaled linearly with coherence and an accumulation process that might include a leak. However, unlike bounded diffusion in the DDM when it was applied to RT data, these models assumed that the accumulation process continued until the stimulus was turned off, at which point the decision was based on the current sign of the decision variable (Gold and Shadlen, 2003; Bogacz et al., 2006). Therefore, these models did not have parameters representing bounds or non-decision times and were fit to psychometric data only (percent correct as a function of both coherence and listening duration).

The model-fitting results suggested only modest, if any, dependence of drift rate or leak on IBI. Drift rate was independent of IBI for all five subjects and depended non-monotonically on IBI for data combined across subjects (Fig. 8*D*). There was also a slight trend for the best-fitting values of drift rate to depend systematically on IBI across subjects (Kruskal−Wallis test for *H*_0_: equal median values per IBI, across subjects, *p* = 0.011^l^). Accumulation leak was not significantly affected by IBI for any of the individual subjects or the data combined across subjects (Kruskal−Wallis test for *H*_0_: equal median values per IBI, across subjects *p* = 0.125^m^; Fig. 8*E*). Thus, the subjects’ decisions improved systematically as a function of the number of tone bursts but were largely independent of the time between bursts.

### Hybrid task

**Figure 9:**
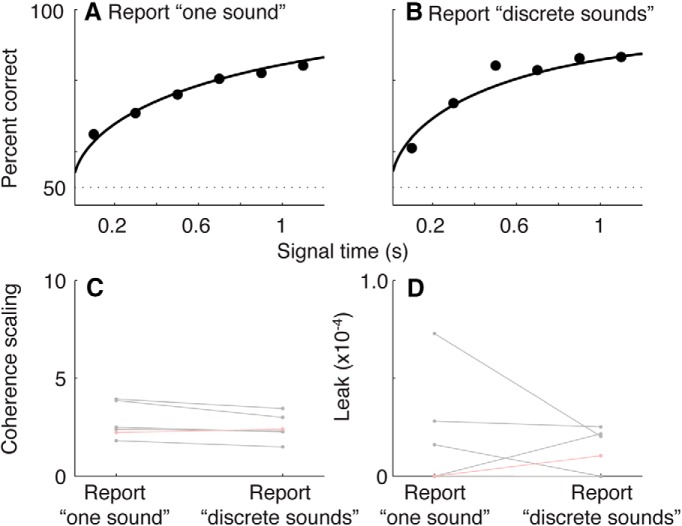
Performance on the hybrid task. ***A***, ***B***, Psychometric data are plotted as a function of listening duration for trials in which the subject reported that the sequence was one sound (***A***) or a series of discrete sounds (***B***). The solid curves are fits from the best-fitting model with two parameters: drift rate and accumulation leak. ***C***, ***D*,** Best-fitting values of drift rate (***C***) and accumulation leak (***D***) plotted as a function of perceptual grouping for fits to data from individual subjects (black) or combined across all subjects (red). The model fits were not improved by fitting the given parameter separately for each IBI condition (likelihood-ratio test, *p* > 0.1^r^ in all cases, Bonferroni-corrected for two parameters).

To test directly the relationship between perceptual grouping and the decision process, subjects participated in the hybrid task, which is a variant of the variable-duration task (Fig. 2*C*). In this task, on each trial, tone-burst sequence was set to each subject’s coherence threshold and IBI threshold (see Materials and Methods, above). At the end of each trial, the subject gave two sequential responses to indicate: (1) perceptual grouping (i.e., one sound or a series of discrete sounds) and (2) sequence direction (i.e., increasing or decreasing). Figure 9 shows psychometric data pooled across all of the subjects, separated into trials in which the subject reported perceiving the sequence as one sound (Fig. 9*A*) or a series of discrete sounds (Fig. 9*B*). Accuracy tended to increase steadily as a function of listening duration in a similar manner for the RT and variable-duration tasks.

To quantify these effects, we fit the data to the same models as those described for the variable-duration task but applied to one sound versus discrete conditions instead of different IBIs (Fig. 9*C*,*D*). These fits indicted that the grouping report did not have any effect on the perceptual decision. For both the drift-rate and accumulation-leak parameters, the data from individual subjects and across subjects were better fit by models that used a single parameter for all trials, as opposed to separate parameters for one sound and discrete reports (likelihood-ratio test, *p* > 0.05^n^). Moreover, when the data were fit separately for the two grouping reports, the resulting best-fitting values did not differ from each other across subjects (Kruskal−Wallis test for *H*_0_: equal median values per grouping judgment, across subjects, *p* > 0.05^o^ for both drift and leak). Thus, the perceptual-grouping judgment did not appear to have a substantial effect on the accumulation efficiency of the sensory evidence.

## Discussion

We examined the relationship between auditory perceptual grouping and decision-making. Our focus was on the role of time in both processes. Specifically, does the temporal manipulation of a stream of acoustic events, which affects its perceptual grouping (Bregman, 1990), affect decisions about its identity? We found that the time interval between sequentially presented tone bursts had strong effects on whether the tone bursts were perceptually grouped as a single sound or heard as discrete sounds (Fig. 3). In contrast, this manipulation did not systematically affect how subjects accumulated information to form a decision about whether frequencies of the tone bursts were increasing or decreasing (Figs. 4-9). Thus, for this task and stimulus, the temporal accumulation of sensory evidence is invariant to the temporal intervals (gaps) between pieces of evidence that affects perceptual grouping.

The effect of time gaps on the time-course of evidence accumulation has also been studied in the visual system (Kiani et al., 2013). In that study, the accumulation of visual evidence was invariant to the temporal gap between pulses of motion evidence for a visual motion direction-discrimination task. We extended those findings by demonstrating that this accumulation invariance is accompanied by a change in the speed−accuracy trade-off that accounts for the different time intervals between the tone bursts. Our results also show that similar principles may govern the temporal dynamics of auditory and visual decisions, despite differences in how the underlying sensory mechanisms process temporal information (Carr and Friedman, 1999; Schnupp and Carr, 2009; Raposo et al., 2012). Below, we discuss the relationships between auditory perceptual grouping and decision-making and then discuss potential neural bases for our findings.

### Temporal dynamics of auditory perceptual grouping and decision-making

Perceptual-grouping cues can affect auditory judgments (Bey and McAdams, 2002; Roberts et al., 2002; Micheyl and Oxenham, 2010; Borchert et al., 2011; Thompson et al., 2011). For example, judgments about the timing differences between auditory stimuli are more accurate when stimuli are grouped into the same auditory stream versus when they are segregated into different auditory streams (Roberts et al., 2002). Similarly, a listener’s ability to detect a deviant tone burst improves when the tone burst is segregated (e.g., by frequency) into a separate auditory stream (Rahne and Sussman, 2009; Sussman and Steinschneider, 2009). In other situations, stream segregation enhances a listener’s ability to identify a tone sequence (Bey and McAdams, 2002).

However, despite evidence for the roles of temporal cues in both auditory grouping and decision-making, little is known about how those roles interact. We addressed this issue by building upon on the rich history of auditory psychophysics and quantitative modeling (Green et al., 1957; Green, 1960; Greenwood, 1961; Luce and Green, 1972; Green and Luce, 1973). Specifically, we applied sequential-sampling models, in particular the DDM, to assess how auditory information presented sequentially over time was used to form a decision about the direction of change of the stimulus frequency.

We used a series of complementary approaches to demonstrate that subjects’ decisions were consistent with a DDM-like process that accumulates sensory evidence over time. First, we fit choice and mean RT data from the RT task directly to several variants of the DDM, all of which effectively described the relationships between stimulus coherence (strength), IBI, and the subjects’ speed−accuracy trade-offs (Figs. 4, 5) (Green and Luce, 1973; Wickelgren, 1977; Palmer et al., 2005; Ratcliff and McKoon, 2008). Second, the subjects’ full RT distributions were also consistent with a rise-to-bound process (Fig. 6), which we fit using a simplified version of DDM models (i.e., the LATER model) that assumes that the rising process is stochastic across trials (as opposed to within trials, for the DDM) and is effective at describing RT distributions across a range of conditions (Carpenter and Williams, 1995; Reddi et al., 2003). Third, analyses of our noisy auditory stimulus indicated that, at least on average, subjects were using information that extended from the beginning of the trial until around the time of the response (Fig. 7). That is, choice selectivity was strongest at the beginning of a trial. Fourth, performance on the variable-duration task increased systematically as a function of listening duration, in a manner consistent with the evidence-accumulation process described by the DDM (Figs. 8, 9).

Our primary result from these analyses was that the accumulation process was invariant to the IBI manipulation. In particular, we found that there was no systematic leak associated with the accumulation process in any of the tested task conditions. Further, the psychophysical kernels were consistent with an evidence-accumulation process with little leak: choice selectivity was strongest at the beginning of a trial and not at the end (which would be expected of a leaky process; Fig. 7). Thus, decisions were consistent with a lossless form of information accumulation (Brunton et al., 2013; Kiani et al., 2013). Moreover, the rate of information accumulation—which was measured in our models as a drift rate and represents the average rate of change of the underlying decision variable—depended only on the coherence of the tone bursts and not the temporal gaps between them. The temporal gaps, instead, affected the speed−accuracy trade-off of the decision (Figs. 5–7). Thus, the evidence-accumulation process was able to use the signals as they arrived without losing information in the intervening gaps, regardless of their duration.

This result was particularly striking in light of the fact that the temporal gaps had a strong effect on the subjects’ percept of the tone-burst stimulus: short gaps gave rise to the percept of a single, grouped sound, whereas longer gaps gave rise to the percept of discrete sounds (Fig. 3). Our findings are, therefore, somewhat surprising, given that previous work has noted an interaction between perceptual grouping and auditory judgments (Bey and McAdams, 2002; Roberts et al., 2002; Micheyl and Oxenham, 2010; Borchert et al., 2011; Thompson et al., 2011). Further work is needed to fully explore these interactions and their relationship to temporal processing. For example, it might be interesting to add nonuniform temporal manipulations to each stimulus sequence (e.g., variable IBIs or tone-burst durations) to get a better sense of how specific timing cues presented at specific times in the stimulus sequence affect both grouping and decision-making.

### Neural basis

Much of auditory perceptual grouping is pre-attentive and has substantive neural signatures in the auditory midbrain and auditory cortex (Carlyon et al., 2001; Fishman et al., 2004; Micheyl et al., 2007; Sussman et al., 2007; Pressnitzer et al., 2008; Shinn-Cunningham, 2008; Winkler et al., 2009). In contrast, auditory decision-making is generally associated with neural mechanisms that are found in the ventral auditory pathway of cortex (Romanski et al., 1999; Romanski and Averbeck, 2009; Rauschecker, 2012; Bizley and Cohen, 2013). In primates, this pathway includes the core and belt fields of the auditory cortex, which project directly and indirectly to regions of the frontal lobe. Because neural activity in these regions, particularly in the belt fields, is not modulated by subjects’ choices, it is thought that this activity represents the sensory evidence used to form an auditory decision but not the decision itself (Tsunada et al., 2011; Tsunada et al., 2012; Bizley and Cohen, 2013; but see Niwa et al., 2012; Bizley et al., 2013). In contrast, frontal lobe activity is modulated by subjects’ choices, consistent with the notion that neural activity in this part of the brain reflects a transition from a representation of sensory evidence to a representation of choice (Binder et al., 2004; Kaiser et al., 2007; Russ et al., 2008; Lee et al., 2009). This hierarchy of information processing is qualitatively similar to that seen in the visual and somatosensory systems (Shadlen and Newsome, 1996; Parker and Newsome, 1998; Gold and Shadlen, 2001; Romo and Salinas, 2001; Romo et al., 2002; Gold and Shadlen, 2007; Hernández et al., 2010).

Thus, our results imply that frontal-mediated decision-making can temporally accumulate evidence from the auditory cortex, independent of how that evidence has been parsed into temporally continuous or distinct groups earlier in the auditory pathway. One possible explanation for our invariance to grouping is that the decision computations use information that is processed separately from the grouping percept. Unfortunately, whereas several studies have reported signatures of grouping in the core auditory cortex (Fishman et al., 2004; Micheyl et al., 2005; Brosch et al., 2006; Selezneva et al., 2006; Elhilali et al., 2009; Shamma and Micheyl, 2010; Fishman et al., 2013; Noda et al., 2013) and representations of the grouping percept in non-core regions of the human auditory cortex (Gutschalk et al., 2005; Gutschalk et al., 2008), neurophysiological studies elucidating where and how perceptual-grouping cues interact with decisions have yet to be conducted. Taken together, however, these aforementioned studies predict that the grouping percept may be mediated in the auditory cortex and frontal activity represents the decision process. A second, alternative explanation is that the decision process may be very flexible and able to efficiently accumulate different forms of noisy evidence under different conditions (Brunton et al., 2013; Kaufman and Churchland, 2013). The degree of this flexibility might depend on the type and quality of the sensory information, memory load, the nature of the environment in which the subject is making the decisions, and other task demands.

## Synthesis

The decision was a result of the Reviewing Editor Tatyana Sharpee and the peer reviewers coming together and discussing their recommendations until a consensus was reached. A fact-based synthesis statement explaining their decision and outlining what is needed to prepare a revision is listed below. The following reviewer agreed to reveal his identity: Mitchell Steinschneider

### Review #1:

The finding that perception of one attribute of sound sequences (i.e., melody, in this case) is independent of whether the component sounds are integrated into one sound stream or heard as independent events. Overall, though, feel this paper is better suited to a more specialized journal.

Summary: For reasons of transparency, this reviewer acknowledges that perceptual modeling is not their expertise. Thus, a detailed critique of the modeling methods will not be provided. Instead, this review will focus on broader issues. In essence, using tone burst sequences as stimuli, the authors clarify the relationship between stream segregation/integration and the determination of whether the melody of a sound sequence rises or falls in frequency. Results indicate that the decision of whether a melody is rising or falling in frequency (one attribute of the sound stream) is independent of whether the individual tones were grouped into one stream, or heard as discrete acoustic events. This reviewer likens this to the perception of a sound melody sequentially played by different instruments. Results are interesting and of potentially great relevance in understanding the neural organization of sound and its discrete attributes. While this reviewer appreciates the significance of the results, the paper is very dense with detailed modeling results, and may not be of major interest to a broad neuroscience audience. In contrast, the paper would be of high interest in a more specialized journal that focuses on hearing and perception. This issue is best left as an editorial decision.

### Major Issues:

1) Despite the intensive modeling, ultimately, the results of the experiments are not a major surprise. A) shorter inter-stimulus-intervals (ISIs) lead to a greater degree of sound sequence integration re longer ISIs, B) Reaction time task: accuracy and speed of response (rising or falling melody) were degraded when the rising and falling sequences were degraded and at longer ISIs, C) subjects decisions with regard to whether the sound sequence was rising (falling) were made based on if the tone frequencies tended to increase (decrease) through much of the trial, D) accuracy of behavioral responses increased with increases in coherence and the number of tone bursts presented, E) accuracy increased with signal duration.

2) Three of the subjects (total # < 10) were authors of the paper and thus not naive listeners. Further, it is quite possible that their responses could be biased toward supporting underlying hypotheses of the work. The data of these subjects should be specifically identified in at least the graphs in order to allow the readership to determine whether these expert listeners significantly biased the results of the experiments.

3) Feedback was always provided to the subjects. Did feedback bias results? Was there a progressive effect of "learning" such that results obtained at the end of a trial block were more accurate and faster than at the beginning? Similarly, was there improved performance in later blocks re earlier blocks?

4) This reviewer is confused as to the validity of the "signal RT" where the silent intervals are subtracted out from the computation of RT. It is stated (line 268) that these periods do not correspond to decision-making time. How can the authors empirically determine that within these intervals the subjects are not involved in the decision-making process? Surely, there will be neural activity in these time periods related to the task. Please explain.

### Minor Issues:

5) Line 91: the phrase "adding or subtracting a fixed‐frequency increment (i.e., 7.5 Hz) to the previous tone‐burst frequency" reads as if the increment was always 7.5 Hz. '(e.g., 7.5 Hz)' would be more accurate.

6) Line 234: The purpose of the Table is not clear to this reviewer.

7) Lines 503-505: The authors cite the midbrain as a major auditory center involved in pre-attentive mechanisms subserving stream segregation/integration, but then go on to cite literature pertaining to the auditory cortex.

8) Figure 4 caption: It is unclear why only the "correct" response data are depicted.

### Review #2:

The paper is relatively well written and appears sound, although I am not well acquainted with the modeling methodology, and some details were unclear. My main concern is that the connection with auditory "grouping" is extremely tenuous. It does not go beyond the observation that tone presentation rate, known to affect decisions as to whether a tone sequence is heard as one event or a sequence of events, also affects the dynamics of decision as to whether the sequence is increasing or decreasing in frequency. This outcome does not seem inconsistent with the hypothesis that both vary with the same stimulus manipulation, but are otherwise unrelated, contrary to what the Abstract suggests (2nd to last sentence).

Two aspects of the design and analysis are puzzling. (a) The models assume a fixed criterion, whereas the stimulus design and order of presentation involve a high degree of variability, so the subject's criterion is likely to vary widely before stabilizing. (b) Performance on each trial is related to the parameters of the statistics of the stimulus, rather than to the statistics of the actual stimuli (an exception is the estimation of the psychophsical kernels). This is a concern particularly as it is not clear whether the conditions are fully balanced (for example in the variable-duration task it is not clear that there are equal numbers of short vs long sequences in each condition). There are many sources of variability, some possibly unnecessary (see details).

Details:

l81: "tone bursts" --> "tones" (burst suggest a cluster of tones)

l85, 86: not intelligible until next paragraph is read

Why do you use "coherence" to manipulate sensory evidence quality? The quality of each sequence will depend on the sequence drawn, introducing a source of variability particularly for response time. Would it not be better to manipulate frequency step size?

l91: With fixed absolute frequency steps the perceptual salience will vary greatly over the range of base frequencies (500-3500 Hz). This is another source of unnecessary variabiliity. Why such a wide range? Why fixed step size? At 3500 Hz the step size (∼0.2%) is similar to the difference limen of selected and highly trained subjects. Most people have much higher thresholds (∼3-10%) so the individual steps (and possibly entire sequences) may be imperceptible for some subjects.

l106: In order to conform to instructions ("be fast but not sacrify accuracy") the subjects must evaluate the difficulty of the task. Given that IBI, coherence, direction, and baseline frequency vary randomly from trial to trial, I imagine that the subjects must take a while to stabilize their criterion, an additional source of variability. The choice is up to the subjects arbitrary decision, and I don't see how one can exclude that this decision depends on IBI in a way unrelated to sensory accumulation.

l110: why is maximum RT equal to maximum sequence duration? What is the intersequence interval, does it depend on the subject's response to the previous sequence?

l130: Were stimulus durations balanced across conditions?

l147: How do you know that coherence threshold was constant across sessions if you do not measure it?

l173: At this point "symmetric bound" is hard to understand. Perhaps precede by a short paragraph explaining the model.

l175: It is not clear to me what is "sensory evidence". Is it pitch, or pitch change, or some estimate of coherence or "signed coherence"? Coherence is a parameter of the stimulus statistics, it is not clear what an internal correlate might look like. Not clear also whether the noise in the model is purely stimulus related or whether it includes a neural component. Why is variance equal to one? Is this a model of a perceptual process, or of the stimulus process? If the latter, why do we need a model since we have perfect knowledge of the stimulus?

l259: In what way is it clear from Fig 4 that choices are not dependent on absolute frequency?

l294: DDM --> DDM parameters

l290: not clear what you mean by "signal". The word is introduced l58 as synonymous with "stimulus", and l267 in the definition of "signal RT". Provide a clear definition.

l299: Not clear what quantities are correlated. Is the high correlation not the trivial consequence of how raw and re-scaled rates are related?

l340: This analysis differs from the usual reverse correlation that averages the stimulus preceding each event in units of time relative to that event. Here the average is over time relative to stimulus onset, regardless of decision time. A decision can't depend on the stimulus that follows it, so some of the data going into this graph is irrelevant (which explains the relaxation to the mean in the later parts). Perhaps there is a better way to quantify what you're describing? Btw it might make more sense to look at dependency on tone-to-tone frequency change rather than frequency (mean subtracted).

l464: As far as I can understand the DDM does not explain grouping.

l476: Obviously they cant use information after their response...

l477: Would one not expect performance to increase with duration for almost any model?

l483: I don't think plots in Fig 7 reflect "choice selectivity". Values reflect a combination of stronger dependency on the initial slope (because post-decision values are irrelevant) and subtaction of the mean over the entire sequence.

l487: Isn't "drift rate" a parameter of the stimulus, rather than of an internal decision variable?

l503 to end: This section is vague and speculative. Any decision based on auditory stimulation must involve all stages of processing up to primary auditory cortex at least, so it does not make sense to assign some to midbrain, others to cortex, etc.
